# Multifocal peliosis hepatis: MR and diffusion-weighted MR-imaging findings of an atypical case

**DOI:** 10.3109/03009730903262118

**Published:** 2010-04-07

**Authors:** Bilal Battal, Murat Kocaoglu, Abdullah Avni Atay, Nail Bulakbasi

**Affiliations:** ^1^Sarikamis Military Hospital, Department of Radiology, Sarikamis, KarsTurkey; ^2^Gulhane Military Medical School, Department of Radiology, Etlik, AnkaraTurkey; ^3^Gulhane Military Medical School, Department of Pediatric Hematology, Etlik, AnkaraTurkey

**Keywords:** DW imaging, MR, peliosis hepatis, restricted diffusion

## Abstract

Peliosis is a rare benign disorder that is characterized by the presence of diffuse blood-filled cystic spaces and can occur in the liver, spleen, bone-marrow, and lungs. We present a 10-year-old boy with Fanconi anemia who presented with peliosis hepatis due to androgen treatment. Magnetic resonance (MR) imaging revealed multiple non-enhancing masses. Some of the lesions revealed fluid-fluid levels and extrahepatic extension on MR images. Diffusion-weighted (DW) imaging showed restricted diffusion. Fluid-fluid levels and extrahepatic extensions are unusual findings for hepatic peliotic lesions. In addition, DW imaging findings of peliosis hepatis have not been reported previously.

## Introduction

Peliosis hepatis is a rare benign entity, which is characterized by the presence of multiple blood-filled lacunar spaces within the liver. This disorder can mimic other hepatic masses, such as hemangioma, hepatocellular carcinoma, abscess, metastasis, adenoma, and focal nodular hyperplasia (1). Imaging findings of peliosis hepatis including ultrasonography (US), computed tomography (CT), and magnetic resonance (MR) imaging have been reported previously (1–7). Here, we present MR and DW-imaging findings of peliosis hepatis with fluid-fluid levels and extrahepatic extension, which developed due to prolonged androgen therapy in a boy with Fanconi anemia. In this report, we describe the DW-imaging findings of peliosis hepatis, for the first time.

## Case report

**Figure 1.**
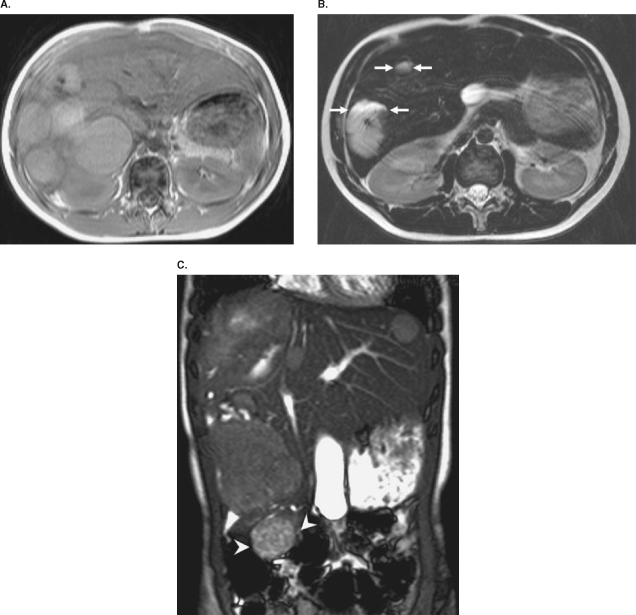
Non-contrast T1-weighted axial magnetic resonance (MR) image (A) shows multiple well defined circumscribed hyperintense masses. On T2-weighted axial MR image (B), lesions demonstrate high signal intensity changes. Two lesions also reveal fluid-fluid level (arrows). On coronal T2-weighted turbo-spin echo MR image (C) one right lobe mass extends outside the liver contours (arrowheads).

**Figure 2.**
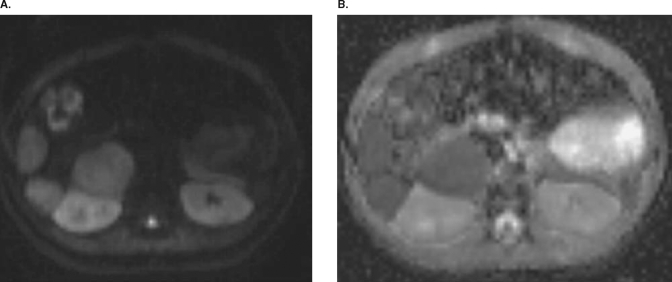
On b = 800 s/mm^2^ (A) diffusion trace image the lesions have high signal intensities. Corresponding apparent diffusion coefficient (ADC) map (B) demonstrates low signal intensities suggesting restricted diffusion.

A 10-year-old boy was admitted to our hospital with Fanconi anemia. After the diagnosis, the patient started treatment with oxymethalone. Eighteen months after the initiation of therapy, liver enlargement and abnormal liver function tests were noted. US revealed multiple solid-appearing hepatic lesions, predominantly hypoechoic, and intralesional small cystic areas. To further characterize the liver lesions, MR imaging was performed by using a 1.5-T MR unit. A routine MR imaging sequence, which included un-enhanced and gadolinium-enhanced axial and coronal T1-weighted fast low angle shot (FLASH) sequences, and axial and coronal T2-weighted turbo-spin echo sequences, were obtained prior to DW imaging. A DW single-shot spin-echo sequence was performed with b-values of 0, 400, and 800 s/mm^2^ during free breathing. An apparent diffusion coefficient (ADC) map was automatically calculated. The lesions were slightly to markedly hyperintense both on T2- and T1-weighted images with no contrast enhancement. Two of the lesions also showed fluid-fluid level on T2-weighted images. In addition, one right lobe mass was extending outside the liver contours (Figure 1). On DW sequences and ADC map the lesions were hyper- and hypointense, respectively (Figure 2). ADC values were variable at the mass lesions (e.g. 0.89–1.06 × 10^-3^ mm^2^/s), and normal-appearing liver had a mean ADC value of 1.21 × 10^-3^ mm^2^/s. Because of the clinical history of the androgen usage, the diagnosis of peliosis hepatis was reached, and the androgen therapy was discontinued.

## Discussion

In peliosis, blood-filled cavitations are seen within lung, bone-marrow, liver, and spleen. Several drugs (such as steroids, oral contraceptives, tamoxifen, and estrogens) and several disease entities (such as malignancies, solid organ transplantations, chronic infections, and *Bartonella* infection) have been reported in the etiology (3,6).

Peliosis hepatis is characterized by the presence of blood-filled cavities in the liver (2). The lesions are almost always multiple and have varying sizes from several millimeters to several centimeters (2–5,8). Parenchymal and phlebectatic variants have been described. Both types may coexist and may result in thrombosis, hemorrhage, and occasionally calcification (8). Hepatocellular necroses, outflow obstruction at the sinusoidal level, and lesions of the sinusoidal barrier have been accused in the etiopathogenesis (8).

The clinical course may range from asymptomatic to a progressive case with liver failure or fatal intra-abdominal bleeding (1,3,6). If clinical and radiological findings are suggestive of peliosis, percutaneous liver biopsy should be avoided because of the significant risk of severe bleeding (2).

Imaging findings are variable and depend on the pathologic presentation and the various stages of the blood component of the lesion. The signal intensity of the lesion on MR imaging largely depends on the age and the status of the blood component. T1-weighted imaging can demonstrate hypo, iso- or hyperintense foci. On T2-weighted images the lesions were reported as hyperintense (4,5). The lesions are typically surrounded by hepatic parenchyma; however, in our case, one of the lesions was atypically extending outside the liver contours and was not completely surrounded by hepatic parenchyma. In addition, we also found fluid-fluid level in two of the lesions. There has been only one reported case of exophytic extension of the hepatic peliotic nodule in the literature (5).

In addition, only one case of fluid-fluid levels was described on CT images previously, by Hiorns et al. (9). Both of these findings are unusual for hepatic peliotic lesions.

With the advent of ultrafast sequences, DW-imaging is now feasible for abdominal studies, and several researchers have used this technique in differential diagnosis of benign and malignant lesions. DW-imaging findings of peliosis hepatis have not been described previously. Although peliosis hepatis is a benign condition, ADC values were lower for than normal-appearing liver, probably due to its content including thrombus and hemorrhage. We speculate that both fluid-fluid levels and low ADC values on MR images were related to old and new blood products in the lesions.

In conclusion, peliosis hepatis must be added in the differential diagnosis of an atypical liver lesion with fluid-fluid levels and restricted diffusion. The use of MR imaging and DW imaging may be useful in the diagnosis of this challenging but rare disease entity. Extension outside the liver contours, fluid-fluid level inside the lesion, and restricted diffusion on MR images may help to differentiate peliosis hepatis from malignant lesions and prevent unnecessary and dangerous interventions.
